# Chemical Composition and Bioactivity of Essential Oil from *Blepharocalyx salicifolius*

**DOI:** 10.3390/ijms19010033

**Published:** 2018-01-04

**Authors:** Fabiana Barcelos Furtado, Bruna Cristina Borges, Thaise Lara Teixeira, Hans Garcia Garces, Luiz Domingues de Almeida Junior, Fernanda Cristina Bérgamo Alves, Claudio Vieira da Silva, Ary Fernandes Junior

**Affiliations:** 1 Institute of Biosciences of Botucatu, Laboratory of Bacteriology, Unesp-São Paulo State University, Botucatu CEP 18618-000, Brazil; fabisbarcelos@hotmail.com (F.B.F.); fe.bergamo@gmail.com (F.C.B.A.); 2 Institute of Biomedical Sciences, Trypanosomatids Laboratory (LATRI), UFU-Federal University of Uberlândia, Uberlândia CEP 38400-902, Brazil; brunacb90@gmail.com (B.C.B.); thaiselara@yahoo.com.br (T.L.T.); silva_cv@yahoo.com.br (C.V.d.S.); 3 Institute of Biosciences of Botucatu, Laboratory of Fungi Biology, Unesp-São Paulo State University, Botucatu CEP 18618-000, Brazil; atiweb@gmail.com; 4 Institute of Biosciences of Botucatu, Laboratory of Phytomedicines, Pharmacology and Biotechnology (PhytoPharmaTech), Unesp-São Paulo State University, Botucatu CEP 18618-000, Brazil; domingues_luiz@hotmail.com

**Keywords:** bicyclogermacrene, globulol, viridiflorol, eudesmol, breast cancer, MDA-MB-231, MCF-10A, *Paracoccidioides brasiliensis*, flow cytometry

## Abstract

Natural products represent a source of biologically active molecules that have an important role in drug discovery. The aromatic plant *Blepharocalyx salicifolius* has a diverse chemical constitution but the biological activities of its essential oils have not been thoroughly investigated. The aims of this paper were to evaluate in vitro cytotoxic, antifungal and antibacterial activities of an essential oil from leaves of *B. salicifolius* and to identify its main chemical constituents. The essential oil was extracted by steam distillation, chemical composition was determined by gas chromatography/mass spectrometry, and biological activities were performed by a microdilution broth method. The yield of essential oil was 0.86% (*w*/*w*), and the main constituents identified were bicyclogermacrene (17.50%), globulol (14.13%), viridiflorol (8.83%), γ-eudesmol (7.89%) and α-eudesmol (6.88%). The essential oil was cytotoxic against the MDA-MB-231 (46.60 μg·mL^−1^) breast cancer cell line, being more selective for this cell type compared to the normal breast cell line MCF-10A (314.44 μg·mL^−1^). Flow cytometry and cytotoxicity results showed that this oil does not act by inducing cell death, but rather by impairment of cellular metabolism specifically of the cancer cells. Furthermore, it presented antifungal activity against *Paracoccidioides brasiliensis* (156.25 μg·mL^−1^) but was inactive against other fungi and bacteria. Essential oil from *B. salicifolius* showed promising biological activities and is therefore a source of molecules to be exploited in medicine or by the pharmaceutical industry.

## 1. Introduction

*Blepharocalix salicifolius* (Kunth) O. Berg is an aromatic species that belongs to the Myrtaceae family, widely distributed in South America. It was reported in Paraguay, Uruguay, Argentina, Bolivia, Ecuador and Brazil [1,2]. In Brazil, it is extensively distributed, being found in the North, Southeast and South regions associated with several climatic conditions (humidity and temperature) [1]. *B. salicifolius* is a tree 10–20 m high and 20–40 cm in diameter. It grows in a straight and cylindrical way, barely tortuous, and with a dark brown bark [3]. Several synonyms are attributed to this species, among them *B. tweediei* and *B. giganteus* [4].

In Brazil, it is known with the name of “Murta”, and is popularly used to treat respiratory diseases, coughs, colds, hypotension, rheumatism, hypoglycemia, diarrhea, leukorrhea, urethritis and bladder diseases [5,6,7,8]. Extracts of this species showed antiparasitic, antifungal, antibacterial, allelopathic, cytotoxic and insecticide effects [2,3,9,10,11,12,13,14,15].

The chemical composition of the essential oil in leaves extracted from this species has been reported and mainly includes 1,8-cineol, α-pinene, limonene, β-pinene, α-terpineol, (*E*)-caryophyllene and linalool [16,17,18,19,20,21,22,23,24,25,26,27]. However, few studies relating to the biological activities of the essential oil from this plant are available. Until now, only fungistatic activity against *Phyllosticta citricarpa* and antitussive, antispasmodic, bronchodilating and cardiac inotropic effects have been described for the essential oil from leaves of this plant [27,28].

Breast cancer has become a great concern, as it is a leading cause of cancer-related deaths for women worldwide [29]. According to the World Health Organization, more than one million cases of breast cancer are reported worldwide annually [30]. High cost, increasing drug resistance, and side effects of current therapeutic approaches are forcing researchers to explore alternative procedures as options to find new chemical entities for cancer treatments [31]. Likewise, microbial pathogens, such as bacteria and fungi, are a major cause of human disease and are among the most concerning threats to public health due to drug-resistant strains [32,33]. A review estimated that continued rise in microbial resistance could lead to 10 million deaths every year by 2050 [34]. Thus, there is a need for the development of new drugs and, due to broad biological action, essential oils or their isolated compounds can inspire novel discoveries. Therefore, the aim of this study was to evaluate in vitro cytotoxic, antifungal and antibacterial activities of essential oils from the leaves of *B. salicifolius*, and to identify their main chemical constituents.

## 2. Results and Discussion

### 2.1. Yield and Chemical Composition of the Essential Oil

The average yield of the essential oil from *B. salicifolius* was 0.86% (*w*/*w*). This is similar when compared to the percentage yield of plants already explored commercially in essential oil production, such as *Ocimum basilicum* (1.24%) and *Thymus vulgaris* (1.05%) [35] or *Eucalyptus citriodora* (1.05%) [36]. 

According to chemical analysis, 29 compounds were identified from the essential oil of the leaves from this species, and sesquiterpene hydrocarbons were predominant (Table 1). Major compounds identified were bicyclogermacrene (17.50%), globulol (14.13%), viridiflorol (8.83%), γ-eudesmol (7.89%), α-eudesmol (6.88%), rosifoliol (4.54%), (*E*)-caryophyllene (4.49%), cubeban-11-ol (4.40%), palustrol (3.83%) and α-pinene (3.22%). 

These results for major compounds are similar to those reported in the literature regarding globulol [17,18], γ-eudesmol and α-eudesmol [22], (*E*)-caryophyllene [21,23] and α-pinene [17,18,20,21,27]. Other terpenes, such as bicyclogermacrene, viridiflorol, rosifoliol, cubeban-11-ol and palustrol, are being described here for the first time as major components of the essential oil in leaves of *B. salicifolius*. Previous studies showed a diverse chemical constitution for the essential oil of this same species, probably because many factors, such as soil characteristics, extraction techniques, climatic characteristics, and others, can affect the quantitative and qualitative constitution of the essential oil [17].

### 2.2. Cytotoxic Activity

The cytotoxic effect of the essential oil was tested in estrogen-receptor positive MCF-7 and estrogen-receptor negative MDA-MB-231 human breast cancer cell lines. Additionally, the human normal breast cell line, MCF-10A, was evaluated. The results of the cytotoxicity assays are shown in Table 2.

The essential oil was toxic against MDA-MB-231 (46.60 μg·mL^−1^) cancer cell lines. MDA-MB-231 cells, which represent an aggressive phenotype, responded more favorably to the cytotoxic effect of the essential oil than the less-aggressive MCF-7 breast cancer cell line. Several difficulties are associated with the treatment of estrogen-receptor negative breast cancer cells because these usually have poor prognosis and hormone therapies are not effective [39], which makes the results found here considered of great relevance. Moreover, this essential oil presented similar results compared to other plant essential oils considered active and with relevant action against this cell type, such as *Decatropis bicolor*, which had a CC_50_ of 53.81 μg·mL^−1^ [40] and *Hedychium spicatum* with values around 65 μg·mL^−1^ [41].

The normal breast cell line, MCF-10A (314.44 μg·mL^−1^), was around sevenfold more resistant to essential oil action than MDA-MB-231 cancer cells. A relationship between normal breast cell lines and breast cancer cell lines was established through the selectivity index (SI) (Table 2) in order to check specificity. A positive SI value is desirable, and represents more selectivity against cancer cells than toxicity to normal cells. The negative value of SI for MCF-7 (−0.21) showed selectivity by normal cells, but the SI for the MDA-MB-231 cells was 0.83, which means selectivity by cancer cells and confirms the potential of this essential oil as a good candidate as a cytotoxic agent.

Against MCF-7, the essential oil did not present cytotoxic action at the tested concentrations. Methanolic extract, chalcones and ursolic acid isolated from the leaves of this species presented cytotoxic activity against such cellular types [9,42]. This suggests that differential content or concentration, especially of polar compounds in *B. salicifolius*, may be related to cytotoxic action against MCF-7 cells.

### 2.3. Flow Cytometry Analysis

**Figure 1:**
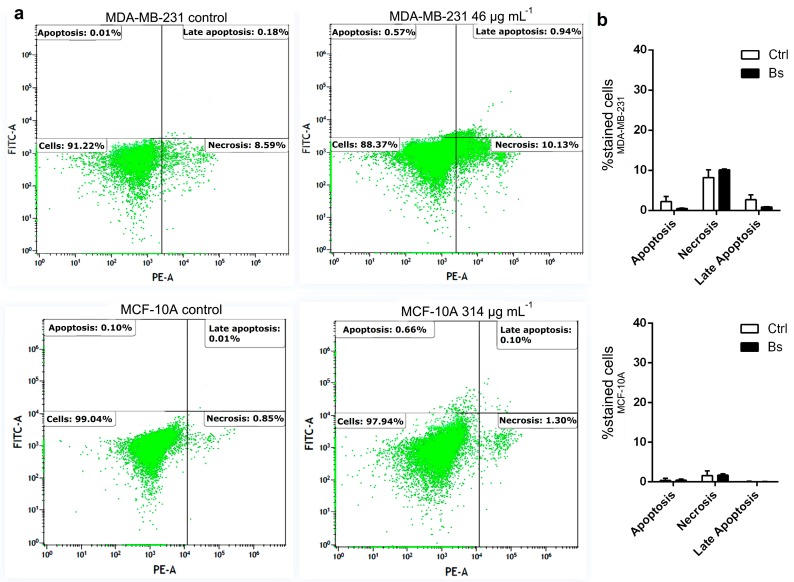
(**a**) Flow cytometry analysis of MDA-MB-231 and MCF-10A cells under action of *B. salicifolius* essential oil; (**b**) Bar diagram demonstrates that there is no difference between control cells and treated cells at the concentration of essential oil determined by cytotoxic activity assays for both cells lines. Ctrl: control; Bs: treatment with *B. salicifolius* essential oil. Data are expressed as mean ± standard deviation for experiments carried out in triplicate. Comparison between bars was performed by two-way ANOVA test followed by Sidak’s test using GraphPad Prism 6.01.

Flow cytometry analysis was performed to investigate if the cytotoxicity effect induced by *B. salicifolius* essential oil is related to pathways that activate cell death. As shown in Figure 1, the treatment with essential oil did not induce cell death in MDA-MB-231 and MCF-10A at the doses of 46 μg·mL^−1^ and 314 μg·mL^−1^, respectively, when compared to control cells. Therefore, the cytotoxicity of this essential oil is not involved with the activation of cell death mechanisms, but with the reduction of the cellular metabolic capacity. The resazurin reduction to verify cell viability may be indicating that there was impairment in cellular metabolism and not necessarily an interruption of electron transport and mitochondrial dysfunction [43].

### 2.4. Antifungal Activity

As shown in Table 3, antifungal activity was not observed against *Candida* spp., *Cryptococcus neoformans* and *Microsporum canis*. A weak action against *Trichophyton mentagrophytes* was found, and *Paracoccidioides brasiliensis* demonstrated a moderate degree of sensitivity to the essential oil based on the minimal inhibitory concentration (MIC) classification adopted by Holetz et al. (2002) [44].

Essential oils with strong antimicrobial activity against yeasts are characterized by a high content of thymol, carvacrol, cymene, linalool or α-pinene [45]. Among the compounds found in the essential oil from *B. salicifolius*, linalool (0.14%) and α-pinene (3.22%) may have exerted some antifungal action against yeasts of *P. brasiliensis*, the causative agent of paracoccidioidomycosis. Few studies related to the action of essential oils against this fungus are available, possibly because it is a mycoses restricted to some countries of Latin America. In addition to previously published studies, this work provides an indication that some terpenes from *B. salicifolius* can be related to inhibition of *P. brasiliensis* growth and this essential oil is therefore a relevant target for future investigation regarding its isolated compounds. Essential oil from *B. salicifolius* showed inhibition with an MIC value lower than already found for other essential oils or isolated compounds tested against this fungus [46,47,48].

Until now, with regard to the antifungal activities of essential oil of the leaves from *B. salicifolius*, only a fungistatic effect against *Phyllosticta citricarpa* was described [28]. Against fungi that are pathogenic to humans, the essential oil of this species had not yet been evaluated. Except for *P. brasiliensis*, the results found for the other fungi indicate that this essential oil was not capable of acting in any mechanism that could provide the fungistatic or fungicidal activity, and therefore, the present terpenes in *B. salicifolius* were not efficient against these species.

### 2.5. Antibacterial Activity

Antibacterial activity of the essential oil from leaves of *B. salicifolius* was determined against some microorganisms of clinical importance, as shown in Table 4.

Essential oils from the leaves of this species has not been investigated previously against any bacterial organism. This study shows that, against the tested bacteria, the essential oil did not have antibacterial activity. However, some studies [2,23] showed activity of extracts from this species against *Staphylococcus aureus* and *Escherichia coli*, which leads to a conclusion that, regarding metabolites from this species, antibacterial activity is dependent on the presence of polar compounds not present in the essential oil. (*E*)-caryophyllene and α-pinene may be responsible for a certain effect against the MRSA and MSSA strains, since other authors have reported antibacterial activity against *S. aureus* when these compounds are in a majority in essential oils [49,50,51,52,53,54,55]. It was observed that the essential oil had greater difficulty in acting against the Gram-negative bacteria. In general, essential oils are more effective against Gram-positive than Gram-negative bacterial strains, because the outer membrane surrounding the cell wall restricts the diffusion of hydrophobic compounds through the lipopolysaccharide covering [56]. Furthermore, the species *Pseudomonas aeruginosa* is generally less susceptible to a diverse range of antimicrobial compounds, including essential oils [57]. This reduced susceptibility has been attributed to the outer membrane and associated properties, such as drug efflux [58,59].

## 3. Materials and Methods 

### 3.1. Plant Material and Extraction of Essential Oil

Leaves of *B. salicifolius* were collected in Botucatu City, Brazil (22°57′55,90″ N 48°24′16,99″ W), on a morning during December 2014. For the purpose of scientific research of the species under study, an authorization to access samples of components of the genetic patrimony (Nº 010621/2015-6) was obtained from the National Council of Scientific and Technological Development (CNPq). The plant was identified by a specialist, and a voucher specimen was deposited in the Herbarium of the Federal University of Uberlândia, under number HUFU 71037. The essential oil was obtained by steam distillation of fresh leaves for 2 h using a distiller designed for essential oil production (model MA480—Marconi). The essential oil was separated from water by decantation, filtered (membrane filter—0.2 μm), stored at low temperature (−10 °C) and protected from light until analysis. The percentage yield was calculated relative to the dried mass of the initial sample and this analysis was performed in triplicate.

### 3.2. Gas Chromatography/Mass Spectrometry (GC/MS) Analysis

Essential oils from *B. salicifolius* leaves were analyzed using a gas chromatograph coupled to a mass spectrometer, model FOCUS ISQ 230ST (Thermo Scientific, Austin, TX, USA), equipped with a TraceGOLD TG-5MS capillary column (Thermo Scientific, 30 m × 0.25 mm × 0.25 μm film thickness). The carrier gas was helium, at a flow rate of 1 mL·min^−1^. Injector and detector temperatures were 220 °C and 240 °C, respectively; the injection volume was 1 μL and split ratio was 1:20. The oven temperature was programmed from 60 °C to 246 °C, at 3 °C/min. The electron impact energy was set at 70 eV and fragments from 40 to 415 *m*/*z* were collected [37].

The identification of the essential oil components was carried out by comparison of the mass spectrum obtained with that stored in the software library (Nist08), and by comparing the calculated arithmetic retention index (AI) according to the equation proposed by Van Den Dool and Kratz (1963) [60] with arithmetic retention index reported in the literature [37,38]. AI calculation was based on retention times of linear alkane standards (C8-C40, Sigma-Aldrich, St. Louis, MO, USA) run under the same operating conditions as previously described. 

Quantitative analysis was carried out in triplicate and the amounts of volatile compounds were calculated using the internal standard method, taking into account the relative response factor (RRF) according to IOFI Working Group on Methods of Analysis [61]. For this purpose, heptanal (Sigma-Aldrich; 0.0092 mg·mL^−1^) was selected as the internal standard. Phellandrene, linalool, caryophylene and nerolidol (Sigma-Aldrich) were selected to represent the response factors for monoterpene hydrocarbons, oxygenated monoterpenes, sesquiterpene hydrocarbons and oxygenated sesquiterpenes, respectively. Analyses for the RRF calculation were completed five times at four concentrations (0.0104, 0.0146, 0.0187 and 0.0229 mg·mL^−1^) [62] and the average response factors were obtained.

### 3.3. Cytotoxic Activity

A sample of the essential oil was solubilized in methanol (Synth, São Paulo, Brazil) and diluted in supplemented Dulbecco’s Modified Eagle Medium (DMEM, Sigma-Aldrich) to form a stock solution of 640 μg·mL^−1^. Cell viability was tested with cell lines from American Type Culture Collection (ATCC, Rockville, MD, USA). MCF-7 (human metastatic adenocarcinoma-estrogen-receptor positive; ATCC HTB-22) and MDA-MB-231 (human metastatic adenocarcinoma-estrogen-receptor negative; ATCC HTB-26) breast cancer cell lines, and the MCF-10A (human mammary epithelial cells; ATCC CRL-10317) normal breast cell line, were selected for cytotoxicity assays. A solution containing 1 × 10^4^ cells in 100 μL of supplemented DMEM was pipetted into each well and the plate was incubated overnight at 37 °C, with a humidified atmosphere and 5% CO_2_, allowing cell adhesion in wells. Once attached and after removal of the culture medium, the stock solution of essential oil was added to the microplate and a serial dilution was performed to achieve concentrations ranging from 4 to 512 μg·mL^−1^. For this analysis, the controls of cell growth, solvent (methanol 3%), samples and negative control (100% lysed cells) were performed. Microplates were incubated for 48 h at 37 °C with a humidified atmosphere and 5% CO_2_. Next, a revealing solution of resazurin (3 mM) diluted in phosphate-buffered saline (PBS) was added to each well [63] and the plate was incubated again for 24 h under the same conditions. Readings of absorbance at 595 nm were performed using a Multiskan GO Microplate Spectrophotometer (Thermo Scientific). Assays were performed in six replicates and the results of absorbance were calculated according to the growth control. The cytotoxic concentration at which 50% of the cells are viable (CC_50_) was calculated by a dose-response graph of nonlinear regression.

In order to check specificity, a relationship between normal breast and breast cancer cell lines was established by the selectivity index (SI) and calculated according to the Equation (1) adapted from Case et al. (2006) [64]:

SI = log (CC_50_ normal cell line/CC_50_ cancer cell line). (1)

### 3.4. Flow Cytometry Analysis

For this analysis, MDA-MB-231 and MCF-10A cells were selected based on results from cytotoxic activity assays. Cells were seeded (5 × 10^6^) in 6-well dishes and treated with essential oil for 48 h at 37 °C at the dose of CC_50_ previously determined by the cytotoxic activity assay. After treatment, the cells were harvested by trypsinisation, washed with PBS and stained with annexin V and propidium iodide (PI) using a FITC Annexin V Apoptosis Detection Kit I (BD Biosciences, San Jose, CA, USA), following the manufacturer’s instructions. The fluorescence was measured in a CytoFLEX Flow Cytometer (Beckman Coulter Inc., Miami, FL, USA) and data analyzed in Kaluza Analysis Software (Beckman Coulter Inc., Miami, FL, USA).

### 3.5. Antifungal Activity

Tested strains were obtained from ATCC and the Laboratory of Fungi Biology, Department of Microbiology and Immunology, São Paulo State University (UNESP-Botucatu Campus). No ATCC strains were molecularly identified and deposited at GenBank database of the National Center for Biotechnology Information (NCBI). The following microorganisms were used in the evaluation of antifungal activity of the essential oil: *Candida krusei* (ATCC 6258), *Candida albicans* (ATCC 36801), *Candida guilliermondii* (ATCC 22017), *Candida parapsilosis* (ATCC 90018), *Candida orthopsilosis* (ATCC 96141), *Candida metapsilosis* (ATCC 96142), *Cryptococcus neoformans* (ATCC 90012), *Paracoccidioides brasiliensis* (strain TLM17LM2, GenBank accession number KX774393, yeast form), *Microsporum canis* (strain RS5, GenBank accession number KT443098) and *Trichophyton mentagrophytes* (ATCC 11480). A microdilution broth susceptibility assay for yeasts and filamentous fungi was performed, as recommended by Clinical and Laboratory Standards Institute [65,66], with some adaptations. Assays were performed on 96-well microplates (Costar, Cambridge, MA, USA) with RPMI-1640 supplemented with glutamine without sodium bicarbonate (Cultilab, Campinas, Brazil). A sample of essential oil was dissolved in dimethyl sulfoxide (DMSO, Synth, São Paulo) and a serial dilution was performed to achieve concentrations ranging from 2.44 to 5000 μg·mL^−1^. The final DMSO concentration was 3% (*v*/*v*). For inoculums, adjustment of yeast (*Candida* spp., *C. neoformans*, *P. brasiliensis*) was performed using microscopic enumeration with a cell-counting hematocytometer (Neubauer chamber, Knittel Gläser, Braunschweig, Germany) from the initial growth at RPMI-1640 medium. *Candida* spp. was grown at 37 °C for 48 h, *C. neoformans* at 37 °C for 72 h and *P. brasiliensis* at 35 °C for 10 days. After counting, the inoculums were adjusted to a final concentration of 1.2 × 10^3^ cell mL^−1^. Filamentous fungi, *M. canis* and *T. mentagrophytes*, were grown on potato dextrose agar (PDA; Oxoid, Basingstoke, Hampshire, UK) for 7 days at 25 °C before counting. Cell counting in a Neubauer chamber was also performed, and microplates were inoculated with a final concentration of 2 × 10^4^ cell mL^−1^. The tested systems were incubated in a humid atmosphere, with agitation, at 37 °C for 48 h for *Candida* spp. and 72 h for *C. neoformans. P. brasiliensis* was incubated for 14 days at 35 °C with agitation and *M. canis* and *T. mentagrophytes* for 10 days at 25 °C without agitation. The experiment was performed in six replicates and the growth inhibition was determined by measuring turbidity of the cultured medium at 530 nm using a spectrophotometer (BioTek, Winooski, VT, USA). Amphotericin B (Sigma-Aldrich) was used as positive control. Controls of sterility of the broth, control culture (inoculum), essential oil and DMSO (3%) were performed.

### 3.6. Antibacterial Activity

Experiments were performed with standard strains from ATCC and clinical isolates (*n* = 6) obtained from the Laboratory of Microbiology, Department of Microbiology and Immunology, São Paulo State University (UNESP-Botucatu Campus). The study was conducted in accordance with the Declaration of Helsinki and the use of isolated microorganisms was approved by the Institutional Committee on Human Research (number 47186415.0.0000.5411-8 March, 2015), according to the ethical principles for medical research involving human subjects, and the strains were submitted to biochemical tests for phenotypic confirmation of the species [67]. The following microorganisms were used: methicillin-resistant *Staphylococcus aureus* (MRSA) (ATCC 33591), methicillin-sensitive *Staphylococcus aureus* (MSSA) (ATCC 25923), *Escherichia coli* (ATCC 43895) and *Pseudomonas aeruginosa* (ATCC 27853). Susceptibility assays were performed according to the Clinical and Laboratory Standards Institute [68] on 96-well microplates (Costar) with Mueller Hinton broth (Difco, Detroit, MI, USA) culture medium. The sample of essential oil was dissolved in DMSO (Synth, 160,000 μg·mL^−1^) and serial dilution was performed to achieve concentrations ranging from 2.44 to 5000 μg·mL^−1^. The final DMSO concentration was 3% (*v*/*v*). Bacterial strains were grown (37 °C for 18–24 h) in brain heart infusion (BHI; Difco) and, after standardization by 0.5 McFarland scale, were inoculated (around 10^5^ cfu·mL^−1^) in wells at concentrations previously prepared. After incubation (37 °C for 18–24 h), the minimal inhibitory concentration (MIC) of each strain was visually recorded after addition of 50 μL of resazurin (0.01%) in respective wells. Polymyxin B (Latinofarma, Cotia, Brazil) was used as a positive control for Gram-negative bacteria and Cephalothin (Latinofarma) for Gram-positive bacteria. Controls of sterility of the broth, culture control (inoculum), essential oil and DMSO (3%) were performed. 

### 3.7. Statistical Analysis

Statistical analysis of the data was performed by analysis of variance (ANOVA) followed by Tukey’s test for analysis of cytotoxic activity and the Kruskal–Wallis test followed by the Student–Newman–Keuls test or Dunn’s test for analysis of minimal inhibitory concentration against microorganisms using SigmaPlot 11.0 software (Erkrath, North Rhine-Westphalia, Germany). For flow cytometry analysis, two-way ANOVA followed by Sidak’s test was performed using the software GraphPad Prism 6.01. Probability values *p* < 0.05 were considered to denote a statistically significant difference.

## 4. Conclusions

In this study, we report cytotoxic and antimicrobial activities of essential oils from the leaves of *B. salicifolius*. This essential oil is a promising cytotoxic agent by its action and selectivity demonstrated here, and is a diverse source of molecules that may have an important role in drug discovery for the treatment of estrogen-receptor negative tumors, which has been a challenge in oncology. Moreover, the antifungal action of this essential oil against *Paracoccidioides brasiliensis*, the causative agent of paracoccidioidomycosis, shows potential to be used in the treatment of this mycosis. Thus, relevant biological activities and high yield of the essential oil from *B. salicifolius* means it is a source of molecules to be exploited for medicine and by the pharmaceutical industry.

## Figures and Tables

**Table 1:** Chemical composition of the essential oil of the leaves from *Blepharocalyx salicifolius.*

Compound	Arithmetic Retention Index (AI) Reference	Arithmetic Retention Index (AI) Calculated	%
*Monoterpene hydrocarbons*			
α-pinene	932 ^a^	925	3.22
β-pinene	974 ^a^	967	0.75
(*E*)-β-ocimene	1044 ^a^	1039	0.26
*Oxygenated monoterpenes*			
linalool	1095 ^a^	1092	0.14
*Sesquiterpene hydrocarbons*			
δ-elemene	1335 ^a^	1329	0.20
α-ylangene	1373 ^a^	1365	0.13
isoledene	1374 ^a^	1370	0.08
β-elemene	1389 ^a^	1384	1.34
α-gurjunene	1409 ^a^	1401	0.41
(*E*)-caryophyllene	1417 ^a^	1411	4.49
NI	-	1419	0.09
NI	-	1426	0.10
α-guaiene	1437 ^a^	1430	0.57
β-barbatene	1440 ^a^	1435	0.12
α-humulene	1452 ^a^	1445	0.47
allo-aromadendrene	1458 ^a^	1453	0.79
NI	-	1467	0.23
germacrene D	1480 ^a^	1473	0.59
4(14),11-eudesmadiene	1487 ^a^	1477	0.24
NI	-	1480	0.19
bicyclogermacrene	1500 ^a^	1488	17.50
germacrene A	1508 ^a^	1496	0.65
γ-cadinene	1513 ^a^	1506	0.09
δ-cadinene	1522 ^a^	1515	0.40
*Oxygenated sesquiterpenes*			
elemol	1548 ^a^	1541	1.39
NI	-	1543	0.40
NI	-	1550	0.59
palustrol	1567 ^a^	1559	3.83
globulol	1582 ^b^	1575	14.13
viridiflorol	1592 ^a^	1583	8.83
cubeban-11-ol	1595 ^a^	1585	4.40
rosifoliol	1600 ^a^	1593	4.54
NI	-	1614	2.27
γ-eudesmol	1630 ^a^	1622	7.89
β-eudesmol	1649 ^a^	1641	3.05
α-eudesmol	1652 ^a^	1645	6.88
			Total (%): 91.26

NI = not identified. ^a^ Adams mass-spectral retention index library [37]; ^b^ NIST: Standard Reference Data [38].

**Table 2:** Cytotoxic concentration (CC_50_ μg·mL^−1^) of essential oil against breast cancer and normal breast cell lines.

Cell Line	*B. salicifolius*	Selectivity index (SI)
MCF-7	>512	−0.21
MDA-MB-231	46.60 ± 8.22 ^a^	0.83
MCF-10A	314.44 ± 60.12 ^b^	-

Six replicates; values are mean ± standard deviation. MCF-7: human metastatic adenocarcinoma; MDA-MB-231: human metastatic adenocarcinoma; MCF-10A: human mammary epithelial cells. Different letters in a column represent significant differences when *p* < 0.05.

**Table 3:** Minimal inhibitory concentration (μg·mL^−1^) for tested fungi.

Fungi Strains	*B. salicifolius*	Amphotericin B
*Candida krusei*	>5000	2
*Candida albicans*	>5000	2
*Candida guilliermondii*	>5000	2
*Candida parapsilosis*	>5000	1
*Candida orthopsilosis*	>5000	0.5
*Candida metapsilosis*	>5000	0.25
*Cryptococcus neoformans*	2500 ^a,^*	0.5
*Paracoccidioides brasiliensis*	156 ^b,^*	0.5
*Microsporum canis*	2500 ^a,^*	2
*Trichophyton mentagrophytes*	625 ^c,^*	1

Six replicates; amphotericin B: positive control. Different letters in a column represent significant differences in the minimum inhibitory concentration between microorganism for the same treatment; * differences when minimal inhibitory concentration value is compared to positive control; *p* < 0.05.

**Table 4:** Minimal inhibitory concentration 90% (μg·mL^−1^) for tested bacteria.

Gram Staining Reaction	Bacterial Strains	*B. salicifolius*	Polymyxin B	Cephalothin
**Gram Positive**	MRSA	4125 ^a,^*	-	2
	MSSA	2062 ^b,^*	-	0.5
**Gram Negative**	*E. coli*	5000 ^c,^*	1	-
	*P. aeruginosa*	>5000	1	-

*n* = 7:1 ATCC and 6 clinical isolates; MRSA: methicillin-resistant *Staphylococcus aureus*; MSSA: methicillin-sensitive *Staphylococcus aureus*. Polymyxin B and cephalothin: positive controls. Different letters in a column represent significant differences in the minimum inhibitory concentration between microorganism for the same treatment; * differences when MIC value is compared to its respective positive control; *p* < 0.05.
